# Substrate for the Myocardial Inflammation–Heart Failure Hypothesis Identified Using Novel USPIO Methodology

**DOI:** 10.1016/j.jcmg.2020.02.001

**Published:** 2021-02

**Authors:** Jakub Lagan, Josephine H. Naish, Kara Simpson, Min Zi, Elizabeth J. Cartwright, Phil Foden, Julie Morris, David Clark, Lindsay Birchall, Jessica Caldwell, Andrew Trafford, Christien Fortune, Michael Cullen, Nazia Chaudhuri, James Fildes, Jaydeep Sarma, Erik B. Schelbert, Matthias Schmitt, Karen Piper Hanley, Christopher A. Miller

**Affiliations:** a Manchester University NHS Foundation Trust, Wythenshawe Hospital, Manchester, United Kingdom; b Division of Cardiovascular Sciences, University of Manchester, Manchester, United Kingdom; c Division of Diabetes, Endocrinology and Gastroenterology, University of Manchester, Manchester, United Kingdom; d Division of Infection, Immunity and Respiratory Medicine, University of Manchester, Manchester, United Kingdom; e Manchester Collaborative Centre for Inflammation Research, University of Manchester, Manchester, United Kingdom; f The Transplant Centre, Manchester University NHS Foundation Trust, Wythenshawe Hospital, Manchester, United Kingdom; g Department of Medicine, University of Pittsburgh School of Medicine, Pittsburgh, Pennsylvania, USA; h UPMC Cardiovascular Magnetic Resonance Center, Heart and Vascular Institute, Pittsburgh, Pennsylvania, USA; i Clinical and Translational Science Institute, University of Pittsburgh, Pittsburgh, Pennsylvania; j Wellcome Centre for Cell-Matrix Research, University of Manchester, Manchester, United Kingdom

**Keywords:** heart failure, magnetic resonance imaging, myocardial inflammation, ultrasmall superparamagnetic particles of iron oxide, DCM, dilated cardiomyopathy, ECV, extracellular volume, HF, heart failure, IQR, interquartile range, LGE, late gadolinium enhancement, LV, left ventricular, MI, myocardial infarction, MRI, magnetic resonance imaging, USPIO, ultrasmall superparamagnetic particles of iron oxide

## Abstract

**Objectives:**

The purpose of this study was to identify where ultrasmall superparamagnetic particles of iron oxide (USPIO) locate to in myocardium, develop a methodology that differentiates active macrophage uptake of USPIO from passive tissue distribution; and investigate myocardial inflammation in cardiovascular diseases.

**Background:**

Myocardial inflammation is hypothesized to be a key pathophysiological mechanism of heart failure (HF), but human evidence is limited, partly because evaluation is challenging. USPIO-magnetic resonance imaging (MRI) potentially allows specific identification of myocardial inflammation but it remains unclear what the USPIO-MRI signal represents.

**Methods:**

Histological validation was performed using a murine acute myocardial infarction (MI) model. A multiparametric, multi-time-point MRI methodology was developed, which was applied in patients with acute MI (n = 12), chronic ischemic cardiomyopathy (n = 7), myocarditis (n = 6), dilated cardiomyopathy (n = 5), and chronic sarcoidosis (n = 5).

**Results:**

USPIO were identified in myocardial macrophages and myocardial interstitium. R1 time-course reflected passive interstitial distribution whereas multi-time-point R2* was also sensitive to active macrophage uptake. R2*/R1 ratio provided a quantitative measurement of myocardial macrophage infiltration. R2* behavior and R2*/R1 ratio were higher in infarcted (p = 0.001) and remote (p = 0.033) myocardium in acute MI and in chronic ischemic cardiomyopathy (infarct: p = 0.008; remote p = 0.010), and were borderline higher in DCM (p = 0.096), in comparison to healthy controls, but were no different in myocarditis or sarcoidosis. An R2*/R1 threshold of 25 had a sensitivity and specificity of 90% and 83%, respectively, for detecting active USPIO uptake.

**Conclusions:**

USPIO are phagocytized by cardiac macrophages but are also passively present in myocardial interstitium. A multiparametric multi-time-point MRI methodology specifically identifies active myocardial macrophage infiltration. Persistent active macrophage infiltration is present in infarcted and remote myocardium in chronic ischemic cardiomyopathy, providing a substrate for HF.

Myocardial inflammation is widely hypothesized to be key driver of heart failure (HF) (1). Persistent myocardial inflammation is thought to have a central role in the development of adverse left ventricular (LV) remodeling following myocardial infarction (MI), as well as in the pathogenesis of dilated cardiomyopathy (DCM) and HF with preserved ejection fraction (1, 2, 3).

However, evaluation of myocardial inflammation in humans is challenging and, as a result, data to support the myocardial inflammation-HF hypothesis in humans are limited. Although circulating inflammatory markers are associated with incident HF, they are not specific to the myocardium (4). Endomyocardial biopsy is not feasible at scale and rarely performed in most centers. Standard cardiovascular magnetic resonance imaging (MRI) (e.g., T1 and T2 relaxation time and extracellular volume [ECV]) and positron emission tomography techniques are not specific for inflammation (5).

Ultrasmall superparamagnetic particles of iron oxide (USPIO), composed of an iron oxide core surrounded by a carbohydrate or polymer coating, passively cross the capillary barrier and appear to be phagocytosed by macrophages. The associated perturbation in magnetic field can be detected with MRI; thus, the method potentially allows identification of active immune cell tissue infiltration and, therefore, inflammation. USPIO-MRI has been applied in cardiovascular diseases; however, histological validation and methodological evaluation of the technique are limited, and as a result, it remains unclear what the USPIO-MRI signal represents (6, 7, 8, 9, 10).

This study aimed to identify where USPIO locate to in myocardium, develop a methodology that differentiates active macrophage uptake of USPIO from passive tissue distribution, and apply the optimized method to investigate myocardial inflammation in a range of acute and chronic human cardiovascular conditions.

## Methods

### Study design

This was a prospective research study. The study comprised 3 parts: 1) A preclinical histological validation study; 2) a human methodological study investigating and developing the USPIO-MRI methodology, which included comparison of R1 and R2* kinetics in active phagocytic cell-rich tissues and active phagocytic cell-poor tissues; and 3) a clinical study applying the optimized USPIO-MRI methodology to investigate myocardial inflammation in a range of acute and chronic cardiovascular conditions.

An ethics committee of the UK National Research Ethics Service approved the clinical studies and written informed consent was obtained from all participants. The work was conducted according to the Helsinki Declaration.

### Preclinical study

See the Supplemental Appendix.

### Methodological investigation: USPIO active uptake versus passive distribution

The study comprised healthy volunteers with no cardiovascular symptoms, no history of medical conditions, no drug allergies, and normal electrocardiogram. Standard cardiac MRI (cine and late gadolinium enhancement [LGE] imaging) performed as part of the study was also normal. Participants were age and sex matched to patients in the clinical study (see the following section).

Participants underwent baseline blood tests (full blood count, renal function, liver function, C-reactive protein, high sensitivity troponin I) and MRI (details follow) followed by intravenous infusion of USPIO (4 mg/kg, ferumoxytol, AMAG Pharmaceuticals) in 0.9% saline. Participants underwent daily MRI for 4 days thereafter. MRI measurements from tissues rich with active phagocytic cells (spleen and liver) were compared with measurements from active phagocytic cell-poor tissues (healthy myocardium).

### Clinical study

Consecutive consenting patients with the following conditions were recruited from Manchester University NHS Foundation Trust:

1. Acute ST-segment elevation MI undergoing primary percutaneous coronary intervention, defined as per standard criteria.
2. Chronic ischemic cardiomyopathy, defined as: established diagnosis per the clinical cardiology team, history of MI at least 10 months before study enrollment, MRI demonstrating an established infarct and LV ejection fraction <50%.
3. Acute myocarditis, defined as: established diagnosis per the clinical cardiology team, acute hospital presentation in keeping with acute myocarditis, elevated serum troponin and C-reactive protein, and MRI typical of myocarditis (as reported by 2 independent experts), including the presence of nonischemic and the absence of ischemic myocardial LGE.
4. DCM, defined as: established diagnosis as per the clinical cardiology team, MRI demonstrating LV ejection fraction <50% with global dysfunction, mid wall septal striae of LGE and no ischemic LGE.
5. Sarcoidosis, defined as: an established multidisciplinary and histological diagnosis of pulmonary sarcoid. All patients also had nonischemic myocardial LGE on MRI. None were thought to have active cardiac sarcoid by their clinical teams.

Exclusion criteria included contraindications to MRI, estimated glomerular filtration rate <50 ml/min/1.73 m^2^, any drug allergy, conditions associated with iron overload, significant liver disease, absolute erythrocytosis/polycythemia, hypotension, bacteremia, pregnancy, breast-feeding, atrial fibrillation, acute HF, and known critical left main stem stenosis/unstable angina.

Participants underwent baseline blood tests and MRI followed by intravenous USPIO infusion. Participants underwent 2 post-USPIO MRI scans.

### MRI

MRI was performed at 1.5-T (Avanto, Siemens Medical Imaging). The baseline MRI scan included standard long- and short-axis steady-state free precession cine imaging. T1 mapping (Modified Look-Locker Inversion Recovery) and T2* imaging (black blood multi-gradient-echo sequence) were acquired at the 4 most basal short-axis slice positions below the LV outflow tract, using the cines as reference, to cover basal and mid-myocardium. Dedicated liver and spleen T1 mapping and T2* imaging were also performed. Following an intravenous bolus of gadolinium contrast agent (0.15 mmol/kg gadoterate meglumine [Dotarem], Guerbet, France), LGE imaging, and repeat T1 mapping were performed (15 min postcontrast for the latter).

USPIO were administered at the conclusion of the baseline MRI. Post-USPIO MRI included long- and short-axis cine imaging, T1 and T2* mapping at slice positions corresponding to those acquired at baseline. No immediate post-USPIO MRI was performed.

### MRI analysis

LV volumes and mass were measured using CVI42 (Circle Cardiovascular Imaging, Canada). T1 maps were generated in Siemens Argus (Siemens Medical Imaging). T2* maps were generated in MatLab v9.0 (The MathWorks, Natick, Massachusetts). Maps were transferred into Horos software (Horos2K v2.2.0, The Horos Project) where endo and epicardial borders, and areas of LGE, were contoured. Two regions of interest were drawn on maps of the liver and spleen, avoiding vessels. Weighted mean voxel relaxation rates, including R1 (longitudinal magnetic resonance relaxation rate) and R2* (transverse magnetic resonance relaxation rate in the presence of static field inhomogeneities) were calculated for whole myocardium, and myocardium with and without LGE where relevant. Similarly, weighted mean voxel relaxation rates were calculated for liver and spleen. Myocardial ECV was calculated as described previously (11).

### Statistical analysis

Data distribution was determined using the Shapiro-Wilk test. Data were summarized using mean ± SD or median (interquartile range [IQR]), and compared using Student's *t*-tests (independent or paired, as appropriate) or nonparametric equivalents. The Student's *t*-tests or nonparametric equivalents were used to compare relaxation times at individual times points and generalized estimating equations, which adjusted for repeated measurements within each subject, were used to compare change in relaxation rates over time between groups. Correlation analysis was performed using Pearson or Spearman correlation as appropriate. Statistical significance was defined as p < 0.05. Analyses were performed using SPSS version 22 (IBM, Armonk, New York).

## Results

### Histological validation

See the Supplemental Appendix.

### Methodological investigation: USPIO active uptake versus passive distribution

Seven healthy subjects were recruited, but 1 was excluded following baseline MRI because of an incidental extracardiac finding. Six subjects completed the protocol. Three were male, mean age 45 ± 18 years. The 4 post-USPIO MRI scans were performed at 19 h (IQR: 16 to 20 h), 50 h (IQR: 43 to 54 h), 75 h (IQR: 69 to 78 h), and 93 h (IQR: 90 to 99 h) following USPIO. No subject experienced an adverse reaction.

**Figure 1:**
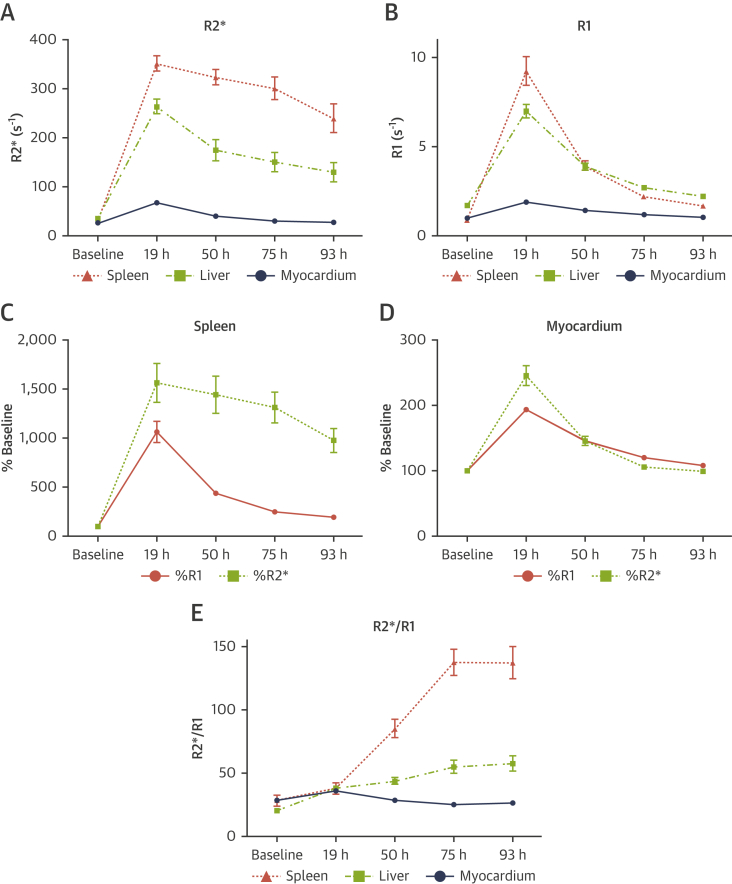
R1 and R2* Behavior in Healthy Subjects Following USPIO **(A)** R2* and **(B)** R1 in spleen, liver, and myocardium. R1 and R2* in **(C)** spleen and **(D)** myocardium, expressed as a percentage of baseline. R2*/R1 ratio in **(E)** spleen, liver, and myocardium. **Error bars** represent SE of the mean. USPIO = Ultrasmall Superparamagnetic Particles of Iron Oxide.

In myocardium, R2* (Figure 1A) and R1 (Figure 1B) both increased shortly after USPIO administration and returned close to baseline by 75 h. In liver and spleen, R2* increased shortly after USPIO administration but remained substantially elevated at 93 h (Figure 1A). Conversely, R1 increased shortly after USPIO administration and returned close to baseline by 93 h (Figure 1B).

As such, tissue R2* and R1 appeared to represent different phenomena following USPIO administration. The time-course of R1 reflected passive interstitial tissue distribution of USPIO, in keeping with the expected plasma elimination of USPIO, but was not sensitive to active phagocytic tissue accumulation. In contrast, although passive tissue distribution also affected R2*, R2* was sensitive to active tissue accumulation. The differences in R2* and R1 are illustrated most clearly in the spleen, a tissue rich with active phagocytic cells, where R2* remained substantially elevated at 75 and 93 h post-USPIO administration but R1 returned close to baseline (Figure 1C). In comparison, in healthy myocardium where there is minimal active phagocytic activity, R2* and R1 behavior were similar (Figure 1D).

As displayed in Figure 1, the differences in R2* and R1 behavior, and hence the ability to detect active tissue USPIO accumulation, were only evident by imaging at multiple time points post-USPIO administration; imaging at a single time point was insufficient. As such, R2* behavior over time was used to evaluate USPIO uptake in the clinical study.

The ratio of R2* to R1 was calculated at each time point to provide quantitative assessment of active tissue USPIO accumulation while taking account of passive tissue distribution. R2*/R1 ratio was found to increase following USPIO administration in liver and spleen, whereas it decreased in myocardium (Figure 1E). The differences in R2*/R1 ratio were most evident on the scan performed at 75 h post-USPIO; thus, this time point was used for comparing R2*/R1 ratio in the clinical study.

Raw R1, R2*, and R2*/R1 data are presented in Supplemental Table 1.

### Clinical study

**Figure 2:**
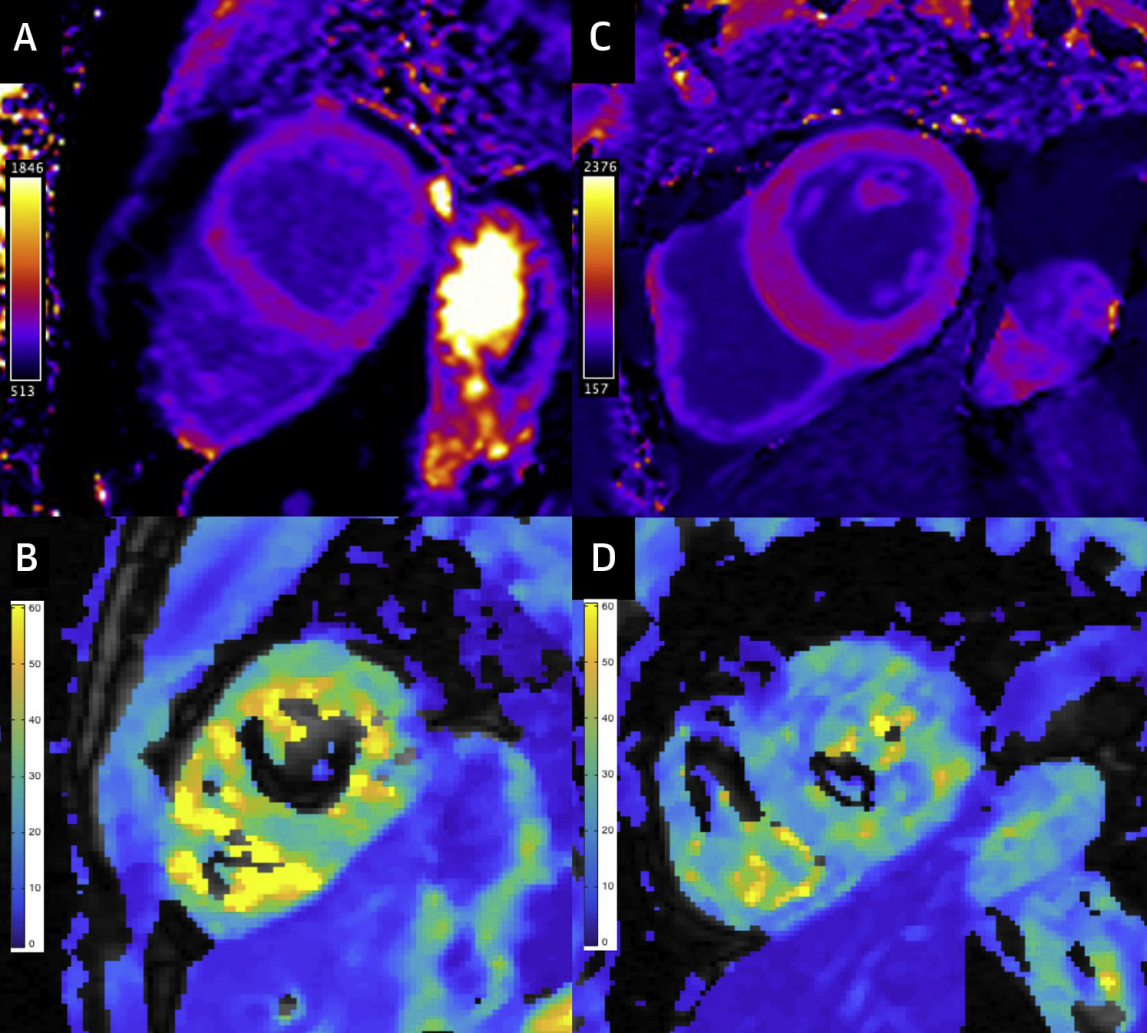
Example Parametric Maps at 75 H Post-USPIO T1 and T2* maps in a healthy volunteer (**A and B**, respectively; R2*/R1 22.6) and in chronic ischemic cardiomyopathy (**C and D**, respectively; R2*/R1 in the infarcted myocardium 32.9; R2*/R1 in the remote myocardium 30.2). USPIO = Ultrasmall Superparamagnetic Particles of Iron Oxide.

**Figure 3:**
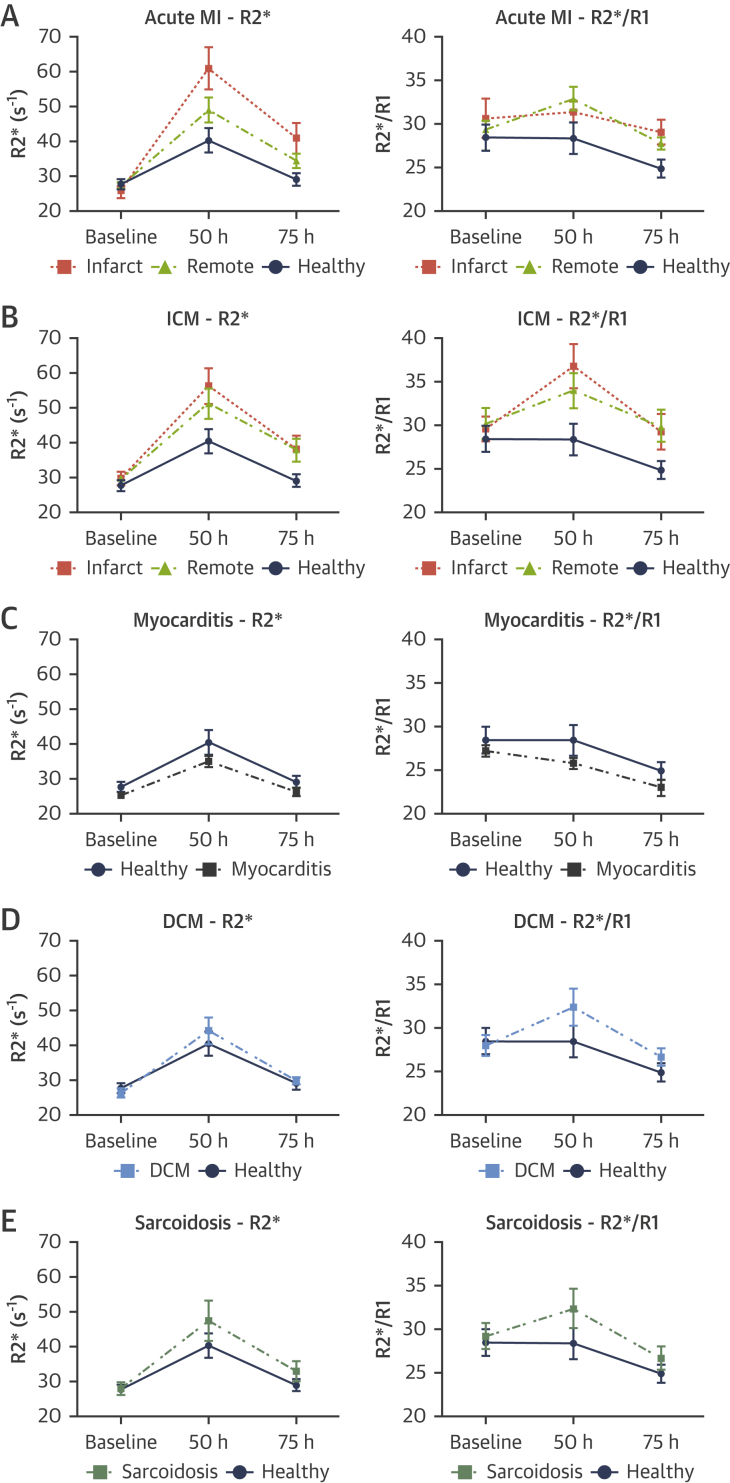
R2* and R2*/R1 Ratio Behavior in Patients With Acute and Chronic Cardiovascular Conditions and Healthy Subjects **(A)** Acute myocardial infarction (MI). **(B)** Ischemic cardiomyopathy (ICM). **(C)** Acute myocarditis. **(D)** Dilated cardiomyopathy (DCM). **(E)** Sarcoidosis. **Error ba**rs represent SEM.

Characteristics of patients in each disease group are presented in Table 1. In light of the validation and methodological findings, MRI was performed before (baseline) and at 2 time points following USPIO (timed to coincide with the 50- and 75-h scans in the healthy cohort). R2* behavior over time and R2*/R1 ratio at 75 h were used to assess for active tissue USPIO accumulation. Healthy volunteers included in the methodological investigation served as a control group. There were no differences in the timing of post-USPIO MRI between each disease and the controls. No patient experienced an adverse reaction. An example of parametric maps at 75 h post USPIO is shown in Figure 2. R2* and R2*/R1 ratio in each disease group are shown in Tables 2 and 3, respectively, and in Figure 3.

**Table 1:** Baseline Characteristics

	Control (n = 6)	Acute MI		ICM		Myocarditis		DCM		Sarcoidosis	
		(n = 10)	p Value	(n = 7)	p Value	(n = 5)	p Value	(n = 5)	p Value	(n = 5)	p Value
Demographics											
Age (yrs)	45 ± 18	60 ± 6	0.10	56 ± 8	0.21	31 ± 13	0.16	57 ± 17	0.26	49 ± 9	0.62
Male	3 (50)	7 (70)	0.61	6 (86)	0.27	5 (100)	0.18	3 (60)	1.00	4 (80)	0.55
Comorbidity											
Hypertension	0 (0)	4 (40)		0 (0)		0 (0)		1 (20)		0 (0)	
TIA/stroke	0 (0)	1 (10)		0 (0)		0 (0)		0 (0)		1 (20)	
Dyslipidemia	0 (0)	2 (20)		0 (0)		0 (0)		1 (20)		1 (20)	
Type 2 diabetes	0 (0)	1 (10)		0 (0)		0 (0)		0 (0)		1 (20)	
Current smoker	0 (0)	7 (70)		1 (14)		1 (20)		0 (0)		0 (0)	
Previous smoker	0 (0)	1 (10)		2 (29)		0 (0)		3 (60)		2 (40)	
Previous PCI	0 (0)	0 (0)		7 (100)		0 (0)		0 (0)		0 (0)	
Previous CABG	0 (0)	0 (0)		2 (29)		0 (0)		0 (0)		0 (0)	
Laboratory findings											
CRP (mg/l)	0 (0-2)	24 (14-41)	<0.01	1 (0-3)	0.35	51 (27-87)	<0.01	1 (0-3)	0.42	3 (2-7)	0.01
WBC (×10^9^/l)	5.6 (4.9-5.6)	11.8 (11.1-13.2)	<0.01	7.5 (6.0-8.2)	0.03	9.6 (8.3-15.0)	<0.01	5.7 (5.3-8.5)	0.47	7.3 (6.6-10.0)	0.01
hsTnI (ng/l)	4 (4-4)	33,370 (4,080-63,184)	<0.01	4 (4-9)	0.17	7,767 (3,448-20,552)	<0.01	7 (5-14)	0.01	4 (4-4)	1.00
Baseline CMR findings											
LV											
LV EDV/BSA (ml/m^2^)	81 ± 17	78 ± 15	0.73	109 ± 21	0.03	83 ± 14	0.83	132 ± 28	<0.01	84 ± 25	0.85
LV ESV/BSA (ml/m^2^)	26 ± 6	39 ± 13	0.04	65 ± 17	<0.01	35 ± 8	0.07	79 ± 23	<0.01	37 ± 13	0.11
LV mass/BSA (g/m^2^)	47 ± 6	69 ± 17	<0.01	66 ± 9	<0.01	66 ± 11	<0.01	64 ± 19	0.08	53 ± 9	0.23
LV EF (%)	68 ± 3	51 ± 7	<0.01	41 ± 5	<0.01	58 ± 5	<0.01	41 ± 6	<0.01	56 ± 4	<0.01
T1 (ms)											
Whole myocardium	1,030 ± 43					1,078 ± 37	0.08	1,067 ± 34	0.15	1,045 ± 41	0.56
Infarcted myocardium		1,193 ± 57	<0.01	1,008 ± 108	0.65						
Remote myocardium		1,076 ± 42	0.05	1,021 ± 34	0.68						
T2* (ms)											
Whole myocardium	37 ± 5					40 ± 2	0.20	39 ± 5	0.51	36 ± 5	0.86
Infarcted myocardium		41 ± 9	0.29	35 ± 6	0.60						
Remote myocardium		37 ± 4	0.87	35 ± 6	0.50						
ECV (%)											
Whole myocardium	26.9 (25.2-28.5)					28.7 (25.8-30.7)	0.20	31.5 (29.7-34.3)	0.01	28.1 (24.6-29.6)	0.72
Infarcted myocardium		44.6 (42.2-52.0)	<0.01	52.2 (44.5-59.2)	<0.01						
Remote myocardium		30.3 (26.6-32.7)	0.08	27.4 (27.1-32.1)	0.57						

Values are mean ± SD, n (%), or median (interquartile range) according to data distribution. The p values refer to the comparison between patient groups and healthy controls.

BSA = body surface area; CABG = coronary artery bypass grafting; CMR = cardiovascular magnetic resonance; CRP = C-reactive protein; DCM = dilated cardiomyopathy; EDV = end-diastolic volume; EF = ejection fraction; ESV = end-systolic volume; hsTnI = high sensitivity Troponin I; ICM = ischemic cardiomyopathy; LV = left ventricular; MI = myocardial infarction; PCI = percutaneous coronary intervention; TIA = transient ischemic attack; WBC = white blood cell.

**Table 2:** Myocardial R2* at Baseline and Following Administration of Ultrasmall Superparamagnetic Particles of Iron Oxide

	Baseline	50 h	75 h	p Value
Control				
Whole myocardium (s^-1^)	27.6 ± 3.6	40.3 ± 8.6	29 ± 4.4	
Acute MI				
Infarcted myocardium (s^-1^)	25.8 ± 6.3	61.0 ± 18.2	40.9 ± 11.5	0.001
Remote myocardium (s^-1^)	27.3 ± 3.2	48.9 ± 11.4	34.3 ± 5.8	0.033
Chronic ICM				
Infarcted myocardium (s^-1^)	29.7 ± 4.9	56.0 ± 13.5	38.2 ± 9.5	0.008
Remote myocardium (s^-1^)	29.7 ± 5.1	51.0 ± 11.5	37.8 ± 9.0	0.01
Myocarditis				
Whole myocardium (s^-1^)	25.2 ± 1.3	34.9 ± 3.6	26.2 ± 2.7	0.461
DCM				
Whole myocardium (s^-1^)	26.2 ± 3.1	43.9 ± 8.6	30.0 ± 2.8	0.096
Sarcoidosis				
Whole myocardium (s^-1^)	28.0 ± 3.9	47.4 ± 13.0	33.0 ± 6.5	0.265

Values are mean ± SD. The p values refer to the comparison of change in R2* over time between patient groups and healthy controls using generalized estimating equations.

DCM = dilated cardiomyopathy; ICM = ischemic cardiomyopathy; MI = myocardial infarction.

**Table 3:** Myocardial R2*/R1 Ratio at Baseline and Following Administration of Ultrasmall Superparamagnetic Particles of Iron Oxide

	Baseline	50 h	75 h	p Value
Control				
Whole myocardium	28.4 ± 3.7	28.3 ± 4.4	24.9 ± 2.6	
Acute MI				
Infarcted myocardium	30.6 ± 6.7	31.4 ± 3.5	29.1 ± 3.8	0.041
Remote myocardium	29.3 ± 3.2	32.9 ± 4.3	27.7 ± 1.9	0.032
Chronic ICM				
Infarcted myocardium	29.6 ± 3.8	36.7 ± 6.7	29.2 ± 5.4	0.095
Remote myocardium	30.2 ± 4.6	33.9 ± 5.3	29.9 ± 4.8	0.042
Myocarditis				
Whole myocardium	27.2 ± 1.7	25.8 ± 1.4	23.0 ± 2.1	0.226
DCM				
Whole myocardium	27.9 ± 2.7	32.3 ± 4.7	26.6 ± 2.2	0.259
Sarcoidosis				
Whole myocardium	29.2 ± 3.3	32.3 ± 5.1	26.7 ± 2.9	0.304

Values are mean ± SD. The p values refer to the comparison of R2*/R1 ratio at scan 3 between patient groups and healthy controls.

Abbreviations as in Table 2.

#### Acute MI

Twelve patients with acute ST-segment elevation MI were recruited but 2 were excluded: 1 because of a USPIO infusion failure and 1 developed an unrelated medical condition. The study therefore included 10 patients; in 5 patients, the LAD artery was the culprit MI artery and in 5 the right coronary artery was the culprit. Mean time between primary percutaneous coronary intervention and baseline MRI was 79 ± 22 h. For logistic reasons, 2 patients underwent 1 post-USPIO MRI only.

On baseline MRI, native T1 and ECV were higher in infarcted myocardium, and were trending to be higher in remote myocardium, compared with healthy control myocardium. There were no differences in native T2*.

R2* behavior over time was different (higher) in infarcted myocardium (p = 0.001), and in remote myocardium (p = 0.033), compared with healthy myocardium. Likewise, R2*/R1 ratio at 75 h was higher in infarcted myocardium (p = 0.041) and in remote myocardium (p = 0.032), compared with healthy myocardium at the same time point.

#### Chronic ischemic cardiomyopathy

Seven patients with chronic ischemic cardiomyopathy were recruited. Median time from MI to enrolment was 1.4 years (IQR: 1.1 to 11.5 years). Mean LV ejection fraction was 41 ± 5%. Four patients had an LAD territory infarct, 2 patients had a right coronary artery territory infarct and 1 patient had infarcts in both territories.

On baseline MRI, native T1 and T2* were no different in infarcted or remote myocardium compared with healthy control myocardium. ECV was higher in infarcted myocardium compared with healthy control myocardium, but ECV in remote myocardium was not.

R2* behavior over time was different (higher) in infarcted myocardium (p = 0.008), and in remote myocardium (p = 0.010), compared with healthy myocardium. R2*/R1 ratio at 75 h was higher in remote myocardium (p = 0.042) and trended toward being higher in infarcted myocardium (p = 0.095), compared with healthy myocardium at the same time point.

Using the R2*/R1 ratio at 75 h post-USPIO in healthy myocardium as a reference, a threshold of 25 had a sensitivity and specificity of 90% and 83%, respectively, for detecting active USPIO uptake in infarcted and remote myocardium in acute MI and chronic ischemic cardiomyopathy (Central Illustration).

**Central Illustration:**
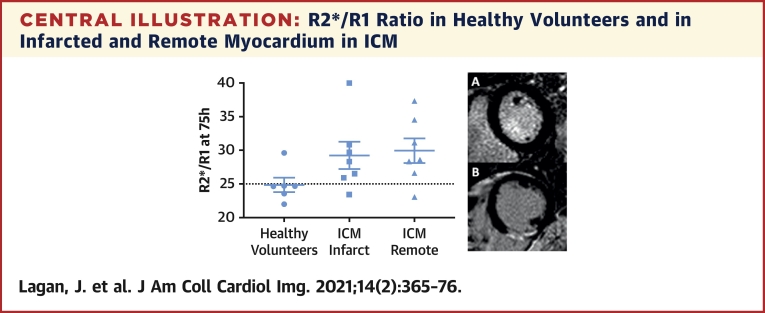
R2*/R1 Ratio in Healthy Volunteers and in Infarcted and Remote Myocardium in ICM Late gadolinium enhancement imaging in **(A)** a healthy volunteer and **(B)** in chronic ischemic cardiomyopathy (ICM).

#### Acute myocarditis

Six patients with acute myocarditis were recruited but 1 patient was excluded because their post-USPIO MRI could not be performed within the required time frame for logistical reasons. Mean time from admission to baseline MRI was 102 ± 52 h.

On baseline MRI, all patients displayed myocardial nonischemic LGE. Regions displaying LGE had higher native T1 and ECV than healthy control myocardium (results not shown). There was a trend towards higher pan-myocardial native T1 and ECV compared with healthy myocardium. There were no differences in native T2*.

Myocardial R2* behavior over time was no different in myocarditis (p = 0.461) compared with healthy myocardium. R2*/R1 ratio was also no different from healthy myocardium (p = 0.226); indeed, in contrast to the other groups, R2* and R2*/R1 ratio were lower than in healthy controls. Findings were the same when analysis was confined to myocardium displaying LGE (results not shown).

#### DCM

Five patients with DCM were recruited. On baseline MRI, all patients displayed myocardial nonischemic LGE. Myocardial ECV was higher in DCM compared with healthy control myocardium and there was a trend toward higher native myocardial T1. There were no differences in native T2*. There was a trend toward a difference in myocardial R2* behavior over time (higher) compared with healthy myocardium (p = 0.096), but R2*/R1 ratio was no different to that in healthy myocardium (p = 0.259).

#### Sarcoid

Five patients with sarcoid were recruited. On baseline MRI, all patients displayed myocardial nonischemic LGE. Native myocardial T1 and T2* were no different compared with healthy control myocardium. Myocardial R2* behavior over time was no different in sarcoid compared with that in healthy myocardium (p = 0.265). R2*/R1 ratio was also no different (p = 0.304).

#### Relationship between native myocardial T1 and USPIO measurements

There were no significant correlations between native myocardial T1 and post-USPIO R2* or R2*/R1 in the whole group or when the analysis was confined to the acute conditions (i.e., acute MI and acute myocarditis) (see Supplemental Results).

## Discussion

Our study has a number of novel findings.

### USPIO are phagocytized by macrophages but are also passively present in the interstitium

USPIO have been used in conjunction with MRI to identify tissue inflammation for a number of years but validation of the technique, including descriptions of the methods and findings, have been limited. Colocalization of USPIO (albeit with a different USPIO preparation [Sinerem]) and macrophages has been demonstrated in vascular tissue and ex vivo (7,10). Stirrat et al. (8) separately demonstrated macrophages and USPIO in infarcted myocardial tissue following USPIO administration in patients with a recent MI undergoing coronary bypass surgery. Our preclinical study demonstrated colocalization of USPIO and macrophages in cardiac tissue for the first time, confirming the ability of USPIO for identifying active cardiac macrophages and myocardial inflammation. Importantly, we also showed that USPIO can be “passively” present in the myocardial interstitium, outside phagocytic cells (see the Supplemental Appendix for details). This is a crucial finding because it demonstrates that the presence of USPIO in tissue is not specific for macrophage activity and therefore, if USPIO are to be used to accurately detect tissue inflammation, the in vivo technique for their detection (i.e., MRI) must be able to differentiate active macrophage USPIO uptake from the passive presence of USPIO in myocardial interstitium.

### USPIO-enhanced multi-time-point multiparametric MRI specifically identifies active myocardial macrophage infiltration

Multiple previous studies, including large clinical trials (7, 8, 9), have measured R2* at a single time point post-USPIO (usually at 24 to 48 h) and described an elevation in post-USPIO R2* in comparison to pre-USPIO R2* as being indicative of tissue inflammation. However, as is evident from Figure 1, R2* is elevated for approximately 75 hours following USPIO administration in healthy myocardium as a result of passive tissue distribution and thus a single elevated post-USPIO R2* is not reflective of active uptake. Other studies have measured R2* at 2 time points post-USPIO (usually 24 and 48 h); however, change in behavior over time has not been assessed, and the timing has been suboptimal (too early) for differentiating active uptake from passive distribution (6). We show that scanning at more than 1 time point following USPIO administration and characterizing R2* behavior over time differentiates active USPIO uptake from passive tissue distribution.

Moreover, after examination of R2* and R1 behavior post-USPIO, we propose a new metric, R2*/R1 ratio, for identifying active myocardial inflammation. R1 is determined by short-range dipolar interactions. R2* is determined by local field gradients. Uniformly dissolved USPIO affect R1 and R2* relaxivity similarly(12). Compartmentalization of USPIO (e.g., with phagocytosis) generates local field gradients and diminishes short-range dipolar interactions (12). Thus, although R1 and R2* are both affected by dissolved USPIO passively distributed through tissue interstitium, R2* is also sensitive to phagocytized USPIO, whereas R1 is not. This is demonstrated most clearly in the spleen, which has high macrophage activity (Figure 1C). R2*/R1 ratio provides a quantitative measurement of active USPIO uptake normalized for background passive tissue distribution; using R2*/R1 ratio at 75 h post-USPIO in healthy myocardium as reference, a threshold of 25 had a sensitivity and specificity of 90% and 83%, respectively, for detecting active USPIO uptake in infarcted and remote myocardium in acute MI and chronic ischemic cardiomyopathy.

For future research, we propose a protocol that includes T1 and T2* mapping at baseline followed by USPIO administration and then repeat T1 and T2* mapping at 50 and 75 h post-USPIO. This will provide R2* behavior over time and R2*/R1 ratio, which, as demonstrated, provide complementary information. Nevertheless, given the effectiveness of R2*/R1 ratio for identifying active macrophage infiltration, it may be that only 1 post-USPIO scan, performed at 75 h, is necessary, which would have logistical and cost benefits.

### Persistent active inflammation in infarcted and remote myocardium in chronic ischemic cardiomyopathy

Through application of the optimized USPIO-MRI method, we demonstrate active inflammation in infarcted and remote myocardium following acute MI, and persistent active inflammation in infarcted and remote myocardium in chronic ischemic cardiomyopathy.

Persistent myocardial inflammation is widely hypothesized as being a key driver of adverse LV remodeling and HF, although there is very little evidence for it in humans (1,13). In preclinical models, active inflammatory cell (including macrophage) infiltration is observed in infarcted and remote myocardium in the chronic phase post-MI (14), and myocardial expression of proinflammatory cytokines is associated with subsequent LV dilatation (15). Indirect evidence in humans includes circulating interleukin-1β levels measured at 2 months post-MI being associated with LV end-diastolic volume at 1 year (16), and, more generally, circulating inflammatory markers being associated with the incidence of, and prognosis in, chronic HF (4). Our study is the first to demonstrate evidence of persistent active macrophage infiltration in infarcted and remote myocardium in the chronic phase (median 1.4 years) post-MI in humans, thus providing direct evidence for the substrate of the hypothesis in humans for the first time. A larger study investigating the relationship between macrophage infiltration and adverse remodeling using USPIO-MRI is now required.

Macrophage infiltration in remote myocardium in the acute phase following MI has been demonstrated in animal models and human tissue (13,17). Increased post-USPIO R2* has been demonstrated in remote myocardium in the acute phase following MI in humans previously. However, because these studies have not performed R2* imaging in conjunction with R1 imaging, imaging has only been performed at a single time point post-USPIO and healthy control data have not been included for comparison, it has been unclear what the elevated R2* represents, with some authors concluding it represents macrophage infiltration (6), whereas others concluding it represents passive tissue distribution (8). By applying our optimized USPIO method, we are able to definitively demonstrate active macrophage infiltration in remote myocardium using USPIO-MRI for the first time. Our findings are in keeping with the limited available positron emission tomography data, although, because fluorodeoxyglucose is avidly taken up by cardiomyocytes, particularly those that are ischemic, the USPIO technique may be advantageous (17).

Although DCM is pathogenically diverse, myocardial immune activation and inflammation are thought to influence the progression of LV dysfunction and adverse outcome (3). Inflammatory infiltrates, including macrophages and T cells, are seen on immunohistological analysis of myocardial tissue obtained at endomyocardial biopsy in approximately one-half of patients with DCM, and are independent determinants of myocardial fibrosis and adverse outcome (3), but myocardial macrophage infiltration has not been demonstrated in vivo in humans. We found a strong trend towards evidence of active macrophage infiltration in DCM. Further investigation is required in a larger cohort.

We did not find evidence of active myocardial macrophage activity in patients with sarcoid, which was in keeping with their clinical status; although patients had pulmonary sarcoidosis and evidence of focal myocardial fibrosis, none was thought to have active cardiac sarcoid by their clinical teams. Future investigation should include patients with active cardiac sarcoid.

In keeping with the recent findings of Stirrat et al. (9), we did not find evidence of active myocardial macrophage activity in myocarditis, despite demonstrating evidence of myocardial edema on native T1 and ECV imaging. This may be because the initial immune response to myocarditis is predominantly lymphocytic. Nevertheless, putting our findings in myocarditis together with those in other pathologies serves to highlight the differing immune response to acute myocardial injury in humans.

Although ferumoxytol has been voluntarily withdrawn from European markets for commercial reasons, it remains available for clinical use elsewhere, including in the United States. Following earlier studies that included 1,164 patients in which the aggregate rate of anaphylaxis was 0.2%, the US Food and Drug Administration in 2015 included a boxed warning for ferumoxytol (Feraheme) highlighting potential fatal and serious hypersensitivity reactions including anaphylaxis, and administration rate was lowered from bolus to infusion. A postmarketing study that included 8,666 patients found the anaphylaxis rate to be only 0.02%, and a number of studies have shown the adverse event rate with ferumoxytol to be no different to that of other intravenous iron products (18,19). A PubMed search using the term “ferumoxytol” identified 600 articles published over the past year (search date September 12, 2019).

### Insight into myocardial inflammation in general

Following on from the observation in myocarditis, our results provide important insight into myocardial inflammation in general. Although the range of potential insults to the myocardium is wide (e.g., ischemic, nonischemic, toxic, mechanical), the response of the myocardium to injury has often been considered to be quite generic, including edema and immune cell infiltration (20). Our results demonstrate that active macrophage infiltration in myocardium does not necessarily go hand in hand with myocardial edema; we found no correlation between native T1, which is determined by tissue water content and is used in the acute setting as a marker of myocardial edema (21), and USPIO uptake. Indeed, our results highlight that the field’s understanding of myocardial inflammation, and “inflammatory cardiac pathologies,” is over simplistic; it is likely that different etiologies are associated with different inflammatory responses, including a variable amount of myocardial edema and spectrum of immune cell activation (e.g., macrophage-dominant vs. lymphocyte-dominant), with differing time courses, which may have a differing impact on patient outcome (22).

### Potential implications for native myocardial iron assessment

Our findings regarding the differential effect of compartmentalized versus “free” iron on R1 may have implications for R1 (or T1) assessment of “native” myocardial iron. T1 has recently been advocated as an alternative to T2* for measuring myocardial and hepatic iron accumulation in conditions associated with iron overload. However, in these conditions, iron is deposited intra- as well as extracellularly; thus, T1 may not accurately reflect total iron levels (23).

### Study Limitations

It was not possible to include preclinical imaging in the validation study. The number of patients with each condition included in the clinical study was small, although we were able to demonstrate significant results despite the small numbers.

## Conclusions

This study confirms the utility of USPIO for identifying active cardiac macrophages but also shows that USPIO can be passively present in the myocardial interstitium, outside of phagocytic cells. A novel multi-time-point multiparametric MRI protocol is developed that specifically identifies active myocardial macrophage infiltration. Application of the new methodology uniquely demonstrates persistent active macrophage infiltration in infarcted and remote myocardium in human chronic ischemic cardiomyopathy, thus providing the first direct evidence in humans of a substrate for the widely held hypothesis that myocardial inflammation is a key driver of HF. The new USPIO-MRI methodology provides the opportunity to investigate the relationship between myocardial inflammation, adverse remodeling and HF in larger studies.

Perspectives **COMPETENCY IN MEDICAL KNOWLEDGE:** Myocardial inflammation is hypothesized to be a key pathophysiological mechanism of HF. A multiparametric multi-time-point USPIO-MRI methodology was developed to specifically identify active myocardial macrophage infiltration. Its application demonstrates persistent active macrophage infiltration in infarcted and remote myocardium in chronic ischemic cardiomyopathy.

**TRANSLATIONAL OUTLOOK:** Investigation of the relationship between myocardial inflammation, adverse remodeling, and HF is required. The new USPIO-MRI technique provides the methodology to achieve this.

## Funding support and Author Disclosures

Dr. Lagan is funded by a Clinical Research Training Fellowship from the British Heart Foundation (FS/17/47/32805). Dr. Karen Piper Hanley is funded by the Medical Research Council Grant (MR/P023541/1). Dr. Miller is funded by a Clinician Scientist Award (CS-2015-15-003) from the National Institute for Health Research. The work was also supported in part by a British Heart Foundation Accelerator award to The University of Manchester (AA/18/4/34221). The views expressed in this publication are those of the authors and not necessarily those of the NHS, the National Institute for Health Research or the Department of Health. The authors recognize the support from AMAG Pharmaceuticals who provided the USPIO. AMAG Pharmaceuticals had no role in study design, data collection, analysis, interpretation or writing of the report. All other authorshave reported that they have no relationships relevant to the contents of this paper to disclose.
