# Supplementary material for: Substrate for the Myocardial Inflammation–Heart Failure Hypothesis Identified Using Novel USPIO Methodology
Source: JACC Cardiovasc Imaging. 2021 Feb;14(2):365–76. doi: 10.1016/j.jcmg.2020.02.001 (PMC7854561; doi:10.1016/j.jcmg.2020.02.001)
Supplement: Supplementary Data [file mmc1.docx]

**Supplemental Appendix**

**Supplemental Methods**

**Preclinical Study**

The preclinical study was performed under approval from the University of Manchester Ethical Review Committee and in accordance with the UK Home Office licence for animal research.

Twelve 13 week old B6 mice were used. Permanent ligation of the left anterior descending (LAD) artery was performed to induce MI in eight mice. After anaesthetic induction with isoflurane (3% isoflurane with 100% oxygen), an intubation tube was inserted into trachea and connected to a rodent ventilator with a stroke volume of 0.2– 0.4 ml/min and respiration rate of 135 breaths/min. Anaesthesia was maintained with 2-3% isoflurane with 100% oxygen. All surgical manipulations were performed under a microscope, maintaining aseptic conditions on a heated surgical pad at 37°C. The pectoral major and minor muscles were retracted. The third intercostal space was exposed and a small hole was made with a mosquito clamp to open the pleural membrane and pericardium. The LAD was ligated with a 7-0 Prolene suture 1 to 2 mm below the tip of the left atrium. Myocardial blanching indicated a lack of perfusion. The chest cavity was closed in two layers (intercostal muscles and skin) with 6-0 silk sutures. The isoflurane was then switched off. Once spontaneous respiration was resumed, the endotracheal tube was removed and the animal placed in a cage on a heating pad until fully conscious. The animals received one dose of buprenorphine (0.3 mg/kg, subcutaneously) 5 minutes before the surgery. Four mice underwent sham surgery which consisted of all the steps listed above, apart from the LAD ligation.

Approximately 24 hours after surgery, USPIO (4mg/kg; ferumoxytol, AMAG Pharmaceuticals, USA) or 0.9% saline were administered intravenously. Four mice undergoing MI and two sham mice received USPIO and four mice undergoing MI and two sham operated mice received saline. Mice were euthanised approximately 72 hours following surgery i.e. 48 hours post USPIO/saline administration. Hearts and livers were harvested, fixed in 4% paraformaldehyde, dehydrated, embedded in paraffin blocks and sectioned.

Myocardium from mid and apical LV underwent immunohistochemical analysis with: (i)anti-Mac-3 antibody with Toluidine Blue counterstain for macrophage detection; (ii)Prussian Blue without counterstain for USPIO detection; (iii)anti-Mac-3 antibody with Prussian Blue without counterstain for simultaneous macrophage and USPIO detection (counterstain was not used because of its similar colour to Prussian Blue; Supplemental Figure 1). Liver tissue underwent immunohistochemical analysis with: (i)anti-F4/80 antibody with haematoxylin counterstain for macrophage detection; (ii)Prussian Blue without counterstain for USPIO detection; (iii)anti-F4/80 with Prussian Blue for simultaneous macrophage and USPIO detection. Please see below for details.

**Immunohistochemistry**

**Liver**

Slides were rehydrated and antigen retrieval was carried out by boiling in 10 mM sodium citrate (pH 6) for 10 minutes. Sections were incubated with Bloxall blocking solution (Vector Laboratories SP-6000) for 10 minutes to quench endogenous peroxidase activity. Slides were then incubated in 2.5% normal goat serum (Vector laboratories S-1012) for 20 minutes followed by overnight incubation in 4ºC with anti-F4/80 antibody (Abcam AB6640; 1:200 solution). On the following day, sections were incubated with anti-rat IgG (ImPRESS Reagent Kit; Vector Laboratories MP-7444) for 30 minutes and then with Diaminobenzidine (DAB) for colour detection. The counterstain was achieved with haematoxylin (Vector Laboratories H-3401) and slides were dehydrated and mounted.

For iron staining, slides were first rehydrated. Slides were then incubated with Prussian Blue staining kit (Clin-Tech 631095) without counterstain for 30 minutes at room temperature. Sections were then dehydrated and mounted. For simultaneous macrophage / Kupffer cells and iron detection, the counterstain step from the anti-F4/80 protocol was substituted with the Prussian Blue stain.

**Heart**

Slides were rehydrated and incubated with 3% H_2_O_2_ for 20 minutes to remove endogenous peroxidase activity. Antigen retrieval was carried out by boiling in 10 mM sodium citrate (pH 6) for 10 minutes. Sections were then incubated with anti-Mac-3 antibody (BD Biosciences 550292; 1:75 solution) overnight at 4ºC. On the following day, slides were incubated first with secondary antibody (anti-rat IgG antibody; Vector laboratories BA-9401) for two hours and then with Streptavidin (Vector laboratories SA-5004). DAB was used for colour detection, sections were counterstained with Toluidine Blue (Fluka Analytical 89640), dehydrated and mounted.

The iron staining was performed as described above. For simultaneous cardiac macrophage and iron detection, the counterstain step from the anti-Mac-3 protocol was substituted with the Prussian Blue stain.

**Results**

One mouse undergoing MI required euthanasia within 24 hours of MI due to severe complications.

Extensive macrophage infiltration was evident in myocardium following MI (Supplemental Figure 2), whereas no macrophages were seen in myocardium following sham surgery (Supplemental Figure 3). In mice undergoing MI that received USPIO, USPIO were seen to be present inside macrophages in myocardium, and in the myocardial interstitial space (Supplemental Figure 2 C-E).

In the liver of mice that received USPIO, iron was seen to be present within macrophages (Supplemental Figure 4).

**Supplemental Results**

**Relationship between native T1 and USPIO indices**

On a whole group analysis, there was no correlation between native T1 and post-USPIO R2* (Scan 2: r=0.04, p=0.773. Scan 3: r=-0.061, p=0.674) or R2*/R1 ratio (r=-0.053, p=0.714). Likewise, when the analysis was confined to the acute pathologies (i.e. acute myocardial infarction and acute myocarditis), there was no correlation between native T1 and post-USPIO R2* (Scan 2: r=0.336, p=0.108. Scan 3: r=0.240, p=0.307) or R2*/R1 ratio (r=0.187, p=0.431).

**Supplemental Figure 1.** Histology sections of mouse hearts stained with MAC-3 (for macrophages) and Toluidine Blue counterstain. (A) and (B) infarcted heart (10x, 20x magnification respectively), shows macrophage infiltration (brown stain; black arrows). Toluidine Blue is seen to stain cardiomyocyte nuclei purple-blue (green arrow), which is a similar colour to Prussian Blue-stained ultrasmall superparamagnetic particle of iron oxide (see Figure 2). (C and D) sham surgery heart (10x, 20x magnification respectively). No macrophages seen. Toluidine Blue-stained cardiomyocyte nuclei visible (green arrow). MI – myocardial infarction.


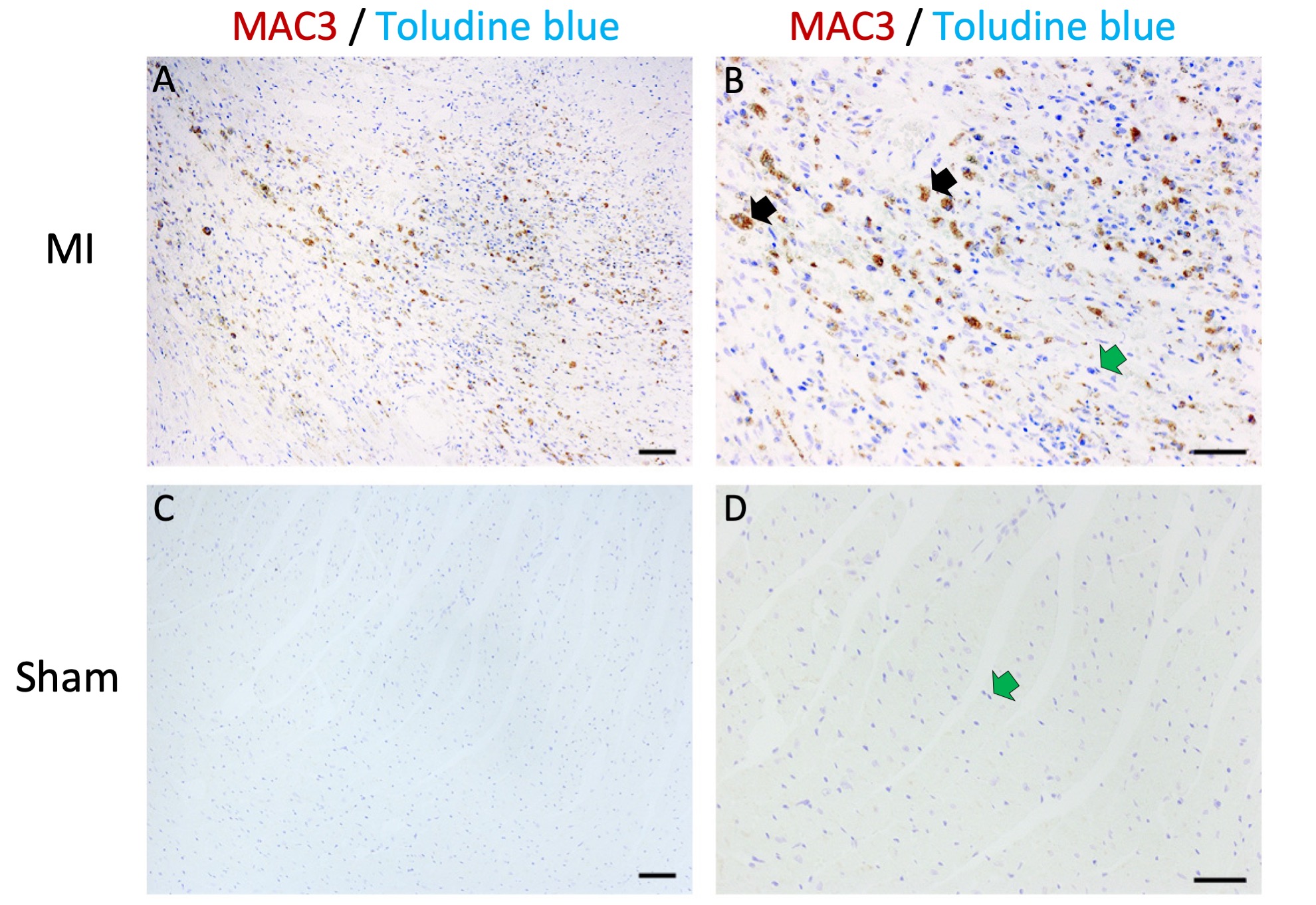


**Supplemental Figure 2. Histology sections of infarcted murine hearts. (**Top row) Following administration of ultrasmall superparamagnetic particles of iron oxide (USPIO). (Bottom row) following administration of saline i.e. no USPIO. (A-B) Complete heart sections. (C) Prussian Blue staining (20x magnification) shows extensive USPIO presence (blue stain; arrows). (D-E) Simultaneous MAC-3 and Prussian Blue staining without counterstain (20x and 40x magnification). USPIO (blue) are seen intracellularly within macrophages (brown; black arrows) and extracellularly in the interstitium (green arrow). (F-G) Complete heart sections. (H) Prussian Blue staining (20x magnification), no USPIO visible. (I-J) Simultaneous MAC-3 and Prussian Blue staining without counterstain (20x and 40x magnification). Macrophages are seen (brown; arrows) but no USPIO are visible. MI – myocardial infarction.


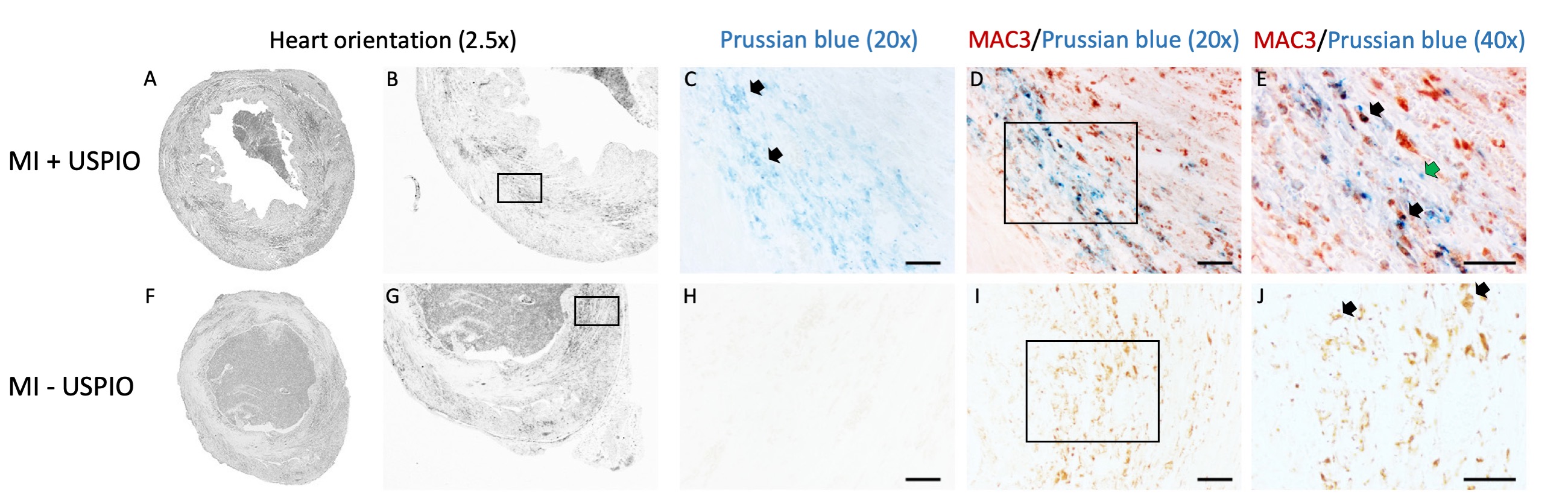


**Supplemental Figure 3.** Heart histology sections from mice undergoing sham surgery. Top row (A – D) ultrasmall superparamagnetic particle of iron oxide (USPIO) administered; bottom row (E – H) no USPIO. (A), (B) Complete heart sections. (C) Prussian Blue staining (20x magnification), no USPIO visible. (D) Simultaneous MAC-3 and Prussian Blue staining without counterstain (20x magnification), neither macrophages or USPIO visible. (E), (F) Complete heart sections. (G) Prussian Blue staining (20x magnification), no USPIO seen. (H) Simultaneous MAC-3 and Prussian Blue staining without counterstain (20x magnification), neither macrophages or USPIO visible.


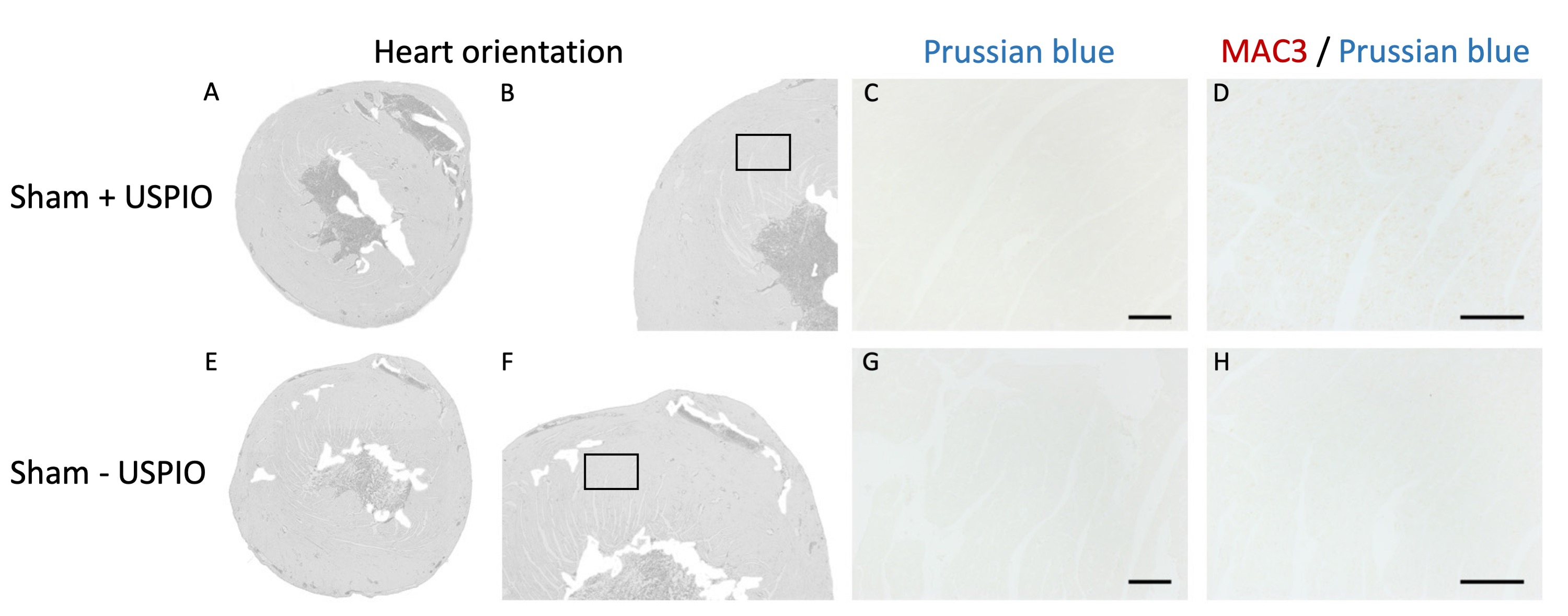


**Supplemental Figure 4.** Liver histology sections from mice receiving ultrasmall superparamagnetic particle of iron oxide (USPIO). (A), (B) F4/80 staining with haematoxylin counterstain (20x, 40x magnification respectively), brown Kupffer cells visible between hepatocytes (arrow). (C), (D) Prussian Blue staining (20x, 40x magnification respectively), blue USPIO visible (arrow). (E), (F) F4/80 and Prussian Blue staining without counterstain, USPIO seen within Kupffer cells (arrow).

**
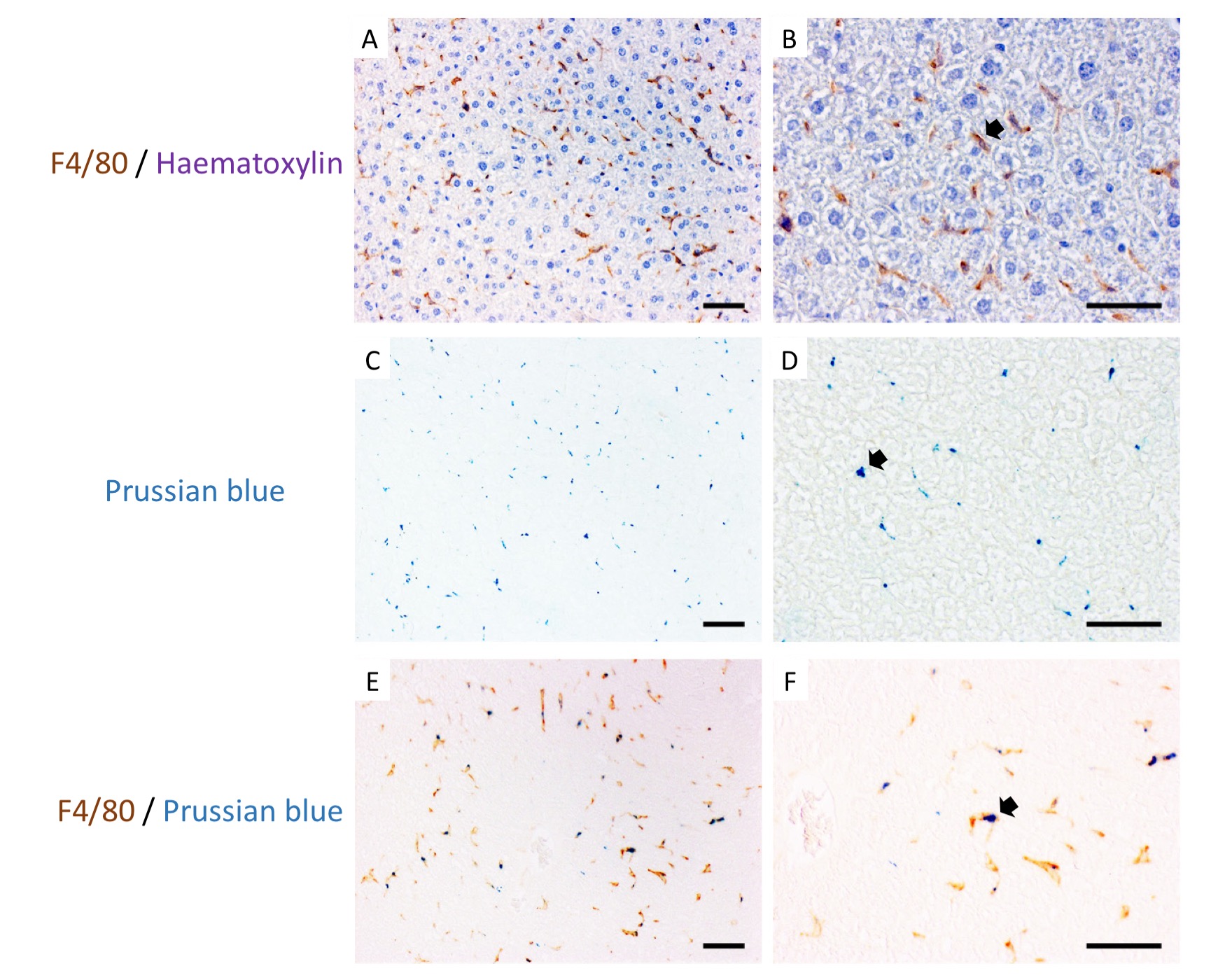
**

**Supplemental Table 1**. **R1, R2* and R2*/R1 ratio in healthy subjects following ultrasmall superparamagnetic particles of iron oxide**

|  | **Baseline** | **19 h** | **50 h** | **75 h** | **93 h** |
| --- | --- | --- | --- | --- | --- |
| **Myocardium** |  |  |  |  |  |
| R1 (s^-1^) | 0.97 ± 0.04 | 1.87 ± 0.12 | 1.42 ± 0.11 | 1.17 ± 0.08 | 1.04 ± 0.04 |
| R2* (s^-1^) | 27.6 ± 3.6 | 67.6 ± 12.3 | 40.3 ± 8.6 | 29.0 ± 4.4 | 27.4 ± 3.6 |
| R2*/R1 | 28.4 ± 3.7 | 36.1 ± 6.5 | 28.3 ± 4.4 | 24.9 ± 2.6 | 26.1 ± 2.9 |
| **Liver** |  |  |  |  |  |
| R1 (s^-1^) | 1.68 ± 0.14 | 6.98 ± 0.93 | 3.92 ± 0.64 | 2.69 ± 0.31 | 2.18 ± 0.17 |
| R2* (s^-1^) | 34.8 ± 7.3 | 263.6 ± 37.0 | 174.0 ± 53.3 | 150.3 ± 48.6 | 129.0 ± 39.4 |
| R2*/R1 | 20.5 ± 2.7 | 37.9 ± 3.3 | 43.8 ± 6.8 | 55.2 ± 12.6 | 57.9 ± 12.0 |
| **Spleen** |  |  |  |  |  |
| R1 (s^-1^) | 0.88 ± 0.05 | 9.25 ± 1.99 | 3.85 ± 0.46 | 2.19 ± 0.20 | 1.70 ± 0.12 |
| R2* (s^-1^) | 25.1 ± 10.9 | 351.4 ± 37.9 | 323.5 ± 38.9 | 300.5 ± 57.3 | 239.4 ± 57.9 |
| R2*/R1 | 28.3 ± 11.0 | 39.2 ± 7.4 | 85.4 ± 17.6 | 137.8 ± 25.5 | 137.3 ± 25.5 |

Data are presented as mean ± standard deviation
